# Analysis of the convergence of the Safety Attitudes Questionnaire and the Hospital Survey on Patient Safety Culture

**DOI:** 10.1590/0034-7167-2021-0379

**Published:** 2022-12-16

**Authors:** Ana Laura Olsefer Rotta, Lucas Paulo de Souza, Manuella dos Santos Garcia Vanti Carvalho, Amanda Pestana da Silva, Andrea Gonçalves Bandeira, Janete de Souza Urbanetto

**Affiliations:** I Pontifícia Universidade Católica do Rio Grande do Sul. Porto Alegre, Rio Grande do Sul, Brazil

**Keywords:** Patient Safety, Quality of Health Care, Organizational Culture, Safety Management, Nursing., Seguridad del Paciente, Calidad de la Atención de Salud, Cultura Organizacional, Administración de la Seguridad, Enfermería., Segurança do Paciente, Qualidade da Assistência à, Saúde, Cultura Organizacional, Gestão de Segurança, Enfermagem.

## Abstract

**Objectives:**

to analyze patient safety culture from nursing professionals’ perception at a university hospital, by assessing the convergence between the Safety Attitudes Questionnaire and the Hospital Survey on Patient Safety Culture.

**Methods:**

a cross-sectional study, with 434 nursing professionals. Data collection took place through the application of both instruments. Descriptive and inferential statistics were used.

**Results:**

in the Hospital Survey on Patient Safety Culture, the “teamwork within the units” dimension was considered a strong area of patient safety. In the Safety Attitudes Questionnaire, the “job satisfaction” and “perception of stress” domains reached the score for a good safety culture. Patient safety culture perception is correlated, in both instruments, with high magnitude.

**Conclusions:**

the two instruments converge towards a similar assessment of patient safety culture.

## RESUMO

**Objetivos:**

analisar a cultura de segurança do paciente a partir da percepção dos profissionais de enfermagem de um hospital universitário, por meio da avaliação da convergência entre o *Safety Attitudes Questionnaire* e o *Hospital Survey on Patient Safety Culture*.

**Métodos:**

estudo transversal, com 434 profissionais de enfermagem. A coleta dos dados ocorreu mediante aplicação de ambos os instrumentos. Utilizou-se estatística descritiva e inferencial.

**Resultados:**

no *Hospital Survey on Patient Safety Culture*, a dimensão “trabalho em equipe no âmbito das unidades” foi considerada uma área forte da segurança do paciente. No *Safety Attitudes Questionnaire*, os domínios “satisfação no trabalho” e “percepção de estresse” atingiram o escore para boa cultura de segurança. A percepção de cultura de segurança do paciente se correlaciona, em ambos os instrumentos, com magnitude elevada.

**Conclusões:**

os dois instrumentos convergem para uma avaliação semelhante da cultura de segurança do paciente.

## RESUMEN

**Objetivos:**

analizar la cultura de seguridad del paciente a partir de la percepción de los profesionales de enfermería de un hospital universitario, evaluando la convergencia entre el *Safety Attitudes Questionnaire y el Hospital Survey on Patient Safety Culture*.

**Métodos:**

estudio transversal, con 434 profesionales de enfermería. La recolección de datos se realizó mediante la aplicación de ambos instrumentos. Se utilizó estadística descriptiva e inferencial.

**Resultados:**

en la Encuesta Hospitalaria sobre Cultura de Seguridad del Paciente, la dimensión “trabajo en equipo dentro de las unidades” fue considerada un área fuerte de la seguridad del paciente. En el *Hospital Survey on Patient Safety Culture*, los dominios “satisfacción en el trabajo” y “percepción de estrés” alcanzaron el puntaje de una buena cultura de seguridad. La percepción de cultura de seguridad del paciente se correlaciona, en ambos instrumentos, con magnitud alta.

**Conclusiones:**

los dos instrumentos convergen hacia una evaluación similar de la cultura de seguridad del paciente.

## INTRODUCTION

The term “safety culture” was first used, in 1986, in the synthesis report on the accident at the Chernobyl nuclear power plant, made by the International Atomic Energy Agency^(1)^, being defined, in 1933, by the Advisory Committee on the Safety of Nuclear Installations, as “product of individual and group values, attitudes, perceptions, competencies, and patterns of behavior that determine the commitment to, and the style and proficiency of, an organization’s health and safety management”^(2-3)^.

Ordinance 529 of April 1, 2013 highlights that the safety culture is based on five characteristics: (a) culture in which all workers, including professionals involved in care and managers, taking responsibility for their own safety, the safety of their colleagues, patients and family members; (b) a culture that prioritizes safety over financial and operational goals; (c) a culture that encourages and rewards the identification, reporting and resolution of safety-related issues; (d) culture that, from the occurrence of incidents, promotes organizational learning-continuous improvement; and (e) culture that provides resources, structure and accountability for the effective maintenance of safety^(4)^.

Several strategies involving safe health care have been developed on the world stage, demonstrating a concern and commitment of professionals and managers with this theme^(5)^. Nonetheless, the disregard for the culture of an institution is worrying, due to its influence on safety promotion. It is important, therefore, that organizations measure and assess their culture to identify needs for improvement to seek safer care^(6)^.

Two instruments are frequently used to measure health safety culture, the Safety Attitudes Questionnaire (SAQ) and the Hospital Survey on Patient Safety Culture (HSOPSC). The SAQ was developed in 2006^(7)^, being adapted to Brazil in 2011^(8)^. The HSOPSC was developed by the Agency for Healthcare Research and Quality (AHRQ) in 2004^(9)^, and adapted to Brazil in 2013^(10)^. Recent studies conducted in Iran^(11)^, Ireland^(12)^, Croatia^(13)^, China^(14)^, Ghana^(15)^, France^(16)^ and the United States of America^(17)^ have also used some of these instruments for safety culture assessment in their hospitals. A survey conducted with health professionals from 68 countries in which the instrument used was the SAQ^(18)^ is also noteworthy.

The safety culture has error prevention as a priority for the entire hospital context, involving from top management to healthcare professionals as well as their patients and families. For an effective culture, it is necessary that health services develop barriers that improve the work process, in addition to a culture of learning, rather than punishment^(19)^.

In a free search in the literature, only two studies were found that investigated the perception of safety culture correlating the HSOPSC and the SAQ, one carried out in the United States of America^(17)^ and another in Brazil^(20)^. Both were focused on Intensive Care Units. Thus, this investigation is justified, with a view to verifying whether, in a hospital environment, the two instruments analyze the perception of patient safety culture in a similar way, making it easier to choose instruments that can bring a related diagnosis. to the topic in the hospital environment. This study was guided by the research question: is there agreement between the SAQ and HSOPSC instruments regarding the perception of nursing about patient safety culture?

## OBJECTIVES

To analyze patient safety culture from nursing professionals’ perception of a university hospital, through convergence assessment between the SAQ and the HSOPSC.

## METHODS

### Ethical aspects

The project was approved by the Research Ethics Committee of the institution in 2017. All participants signed the Informed Consent Form and received a copy.

### Study design, period, and location

This is a cross-sectional study, carried out in a large university general hospital, located in the city of Porto Alegre, Grande do Sul. To guide this study, the Strobe checklist was adopted. The data collection period was from August 2018 to July 2019, in the morning, afternoon, evening and intermediate shifts (morning and afternoon or afternoon and night), during professionals’ workday.

### Population, sample; inclusion and exclusion criteria

The population consisted of 1,475 nursing professionals (nurses, nursing technicians and assistants). The remaining health workers were not included in this investigation. However, this group is representative, as nursing professionals represent the largest category of professionals responsible for patient care and play a vital role in the implementation of quality and safety measures^(21)^. All professionals, according to the researched institution’s orientation, can report on safety incidents.

All the nursing professionals approached, aged 18 years or older, who agreed to participate were included, with the exception of those who had the exclusion criterion of working at the institution for less than six months (since the short length in the hospital can be considered bias, due to lack of knowledge of organization and institutional culture). The minimum sample calculated for this study would be 428 professionals, based on the sample calculation performed, considering a sampling error of 4%, an estimated percentage of 50% and an error significance level of 5%.

Through a non-probabilistic sampling, 655 professionals were approached and invited to participate in the research. Seventy-two professionals with less than six months of admission and 86 who did not return the data collection instrument (DCI) were excluded. Due to problems identified in completing the answers to the instruments, 12 professionals were also excluded. Fifty-one professionals refused to participate in the study. Thus, the sample of this research was composed of 434 professionals.

### Study protocol

The data collection team was composed of researchers, all previously trained. Data were collected through the application of a data collection instrument, filled in by participants, consisting of demographic variables (age and sex), employment (position, work shift, workload, unit, length of training in the position, job tenure and in relation to professional performance in another location), and issues related to patient safety culture from the application of HSOPSC and SAQ.

The HSOPSC seeks to obtain the perception of culture through 12 dimensions. These address key points regarding the organization’s beliefs and norms, values, communication, leadership, management, and communication of adverse events ^(10)^. The dimensions add up to 50 items in total. Thus, 44 are related to the specific questions about safety culture and the other six items, related to personal information^(9)^. Of the items in the instrument, 33 were answered using a five-point Likert scale, expressing the degree of agreement from “totally disagree” to “totally agree”. Nine items, in turn, using a five-point frequency scale, from “never” to “always”. Professionals were also able to assign a grade to patient safety in their unit/work area, with options ranging from “excellent” to “very poor”. Finally, in relation to the item referring to the number of events reported in the last 12 months, there were options from “none” to “11 to 20 events”^(10)^.

The SAQ is structured in two parts. The first has 41 items that are related to seven domains. The response of each item follows a five-point Likert scale, from “strongly disagree” to “strongly agree”, adding the “not applicable” option. The second part collects professional data^(8)^. The score is ordered in “strongly disagree”, equivalent to zero points, “partially disagree” - 25 points, “neutral” - 50 points, “partially agree” - 75 points and “strongly agree” - 100 points. However, items 2, 11 and 36 are reversed. The sum of the responses of the items of each domain is then calculated by dividing the result by the number of items of each domain^(8)^.

### Analysis of results, and statistics

The perception of patient safety culture, according to the HSOPSC, in the Brazilian version, occurred through the percentage of positive responses for each dimension, using the following formula: [number of positive responses to the item of the assessed dimension/total number of valid responses to the items of the assessed dimension (positive, neutral and negative, excluding missing data)] x 100 The percentage of positive responses represents a positive reaction to patient safety culture, allowing the identification of strong and fragile areas for patient safety. “Strong areas of patient safety” are those whose items written positively obtain 75% of positive responses or those whose items written negatively obtain 75% of negative responses. Similarly, “fragile areas of patient safety” that require improvement are those whose items achieve 50% or less positive responses^(10)^.

The descriptive analysis of the SAQ consisted of the mean of the responses and the calculation of professionals’ responses to the 41 items. The formula used was [(m-1) x 25], where m is the mean of the items of the domain in question. Analysis was performed by the overall SAQ and the domains. The score of zero represents the worst perception, and the score of 100, the best. Therefore, values equal to 75 points or greater indicate a strong agreement of professionals regarding issues related to patient safety^(8)^.

Data were entered into Excel spreadsheets, by double entry, with subsequent verification and correction of inconsistencies. Analysis was performed using the Statistical Package for the Social Sciences (SPSS), version 18.0 for Windows. Descriptive statistics - absolute and relative frequencies, measures of central tendency (mean and median) and variability (breadth and standard deviation) - were used to characterize the study participants. To analyze continuous variable distribution symmetry, the Kolmogorov-Smirnov test was used and, whenever in the presence of asymmetric data, in the description, median and breadth.

In inferential statistics, Pearson’s or Fisher’s chi-square test (for association analysis) and Pearson correlation test (for correlation analysis between instruments) were used, adopting a significance level of 5%. As for the magnitude of correlation, low magnitude (0.10 to 0.30), moderate magnitude (0.30 to 0.50) and high magnitude (0.50 to 1)^(22)^ were considered.

The internal consistency of responses to the two instruments was measured by Cronbach’s alpha coefficient, adopting the classification of very low (alpha ≤ 0.30), low (0.30 < alpha ≤ 0.60), moderate (0.60 < alpha ≤ 0.75), high (0.75 < alpha ≤ 0.90) and very high (alpha > 0.90)^(23)^. Age, job tenure and length of training in the position were dichotomized by median due to abnormal data distribution (Kolmogorov-Smirnov < 0.005).

## RESULTS

Female professionals comprised 82.9% (n=360) of the sample. Moreover, 51.6% (n=223) of professionals were aged up to 34 years (minimum of 18 and maximum of 64 years), and 48.4% (n=209) were 35 years of age or older. Regarding work variables, professionals in the position of nursing technician/assistant comprised 74.9% (n=325) of participants. The work units represented were inpatient units (50.5%; n=219), Intensive Care Units (29.0%; n=126), surgical center and obstetric center (10.1%; n=44), emergency units (8.3%; n=36) and other units (2.1%; n=9).

The night shift was composed of 49.1% (n=213) afternoon (28.3%) (n=123), morning (16.1%) (n=70) and intermediate (6.5%) (n=28) professionals. The workload of 12 hours comprised 49.5% (n=215), of 6 hours, 49.1% (n=213), and the rest of the sample consisted of workloads from 8 to 10 hours, with 1.4% (n=6). Regarding job tenure, 52.1% (n=226) of the sample had worked for up to three years and 47.9% (n=208) had worked for four years or more (minimum of six months and maximum of 33 years). Regarding training in the position, 52.5% (n=228) had training up to seven years and 47.5% (n=206), eight years or more (minimum of seven months and maximum of 33 years). Finally, 88% (n=382) of professionals did not work at another institution.

The HSOPSC and SAQ instruments obtained high internal consistency by Cronbach’s alpha coefficient of 0.828 and 0.757, respectively. Table 1 presents the HSOPSC descriptive data (by dimensions and overall).

**Table 1:** Analysis of central tendency and variability measures and frequencies of classifications of fragile areas and strong areas of patient safety from the Hospital Survey on Patient Safety Culture, Porto Alegre, Rio Grande do Sul, Brazil, 2018/2019 (n = 434)

HSOPSC	Median	Minimum	Maximum	n	Fragile areas of patient safety n (%)	Strong areas of patient safety n (%)
HSOPSC^*^ dimensions						
DIM1^†^ - Teamwork within units	75.00	0	100.00	434	172 (39.6)	262 (60.4)
DIM2 - Supervisor/manager expectations & actions promoting safety	50.00	0	100.00	434	248 (57.1)	186 (42.9)
DIM3 - Organizational learning-continuous improvement	66.66	0	100.00	300	165 (55.0)	135 (45.0)
DIM4 - Feedback and communication about error	33.33	0	100.00	325	231 (71.1)	94 (28.9)
DIM5 - Communication openness	33.33	0	100.00	313	246 (78.6)	67 (21.4)
*DIM6 - Staffing*	50.00	0	100.00	433	334 (77.1)	99 (22.9)
DIM7 - Nonpunitive response to error	0	0	100.00	379	366 (96.6)	13 (3.4)
DIM8 - Hospital management support for patient safety	33.33	0	100.00	336	234 (69.6)	102 (30.4)
DIM9 - Teamwork within Units	25.00	0	100.00	434	360 (82.9)	74 (17.1)
DIM10 - Hospital handoffs & transitions	25.00	0	100.00	434	351(80.9)	83 (19.1)
DIM11 - Perceptions of management	25.00	0	100.00	433	339 (78.3)	94 (21.7)
DIM12 - Frequency of event reporting	33.33	0	100.00	389	218 (56.0)	171 (44.0)
Overall HSOPSC	44.44	2.08	95.14	291	257 (88.3)	34 (11.7)

* *HSOPSC - Hospital Survey on Patient Safety Culture.*

† *DIM - Dimension. Note: in the classification between fragile and strong areas of the HSOSPC, “n” does not reach the total sample because the score between 50% and > 75% is not classified.*

In the HSOPSC, with regard to the grade that professionals attributed to patient safety in their area/work unit, 47% (n=204) of professionals considered patient safety as “regular”, 40.3% (n=175) as “very good”, 5.5% (n=24), “poor” 5, 3% (n=23), “excellent” and 1.9% (n=8), “very poor”. Regarding the question about the number of reporting made in the last 12 months, 48.2% (n=209) of professionals answered that they did not report any, 19.8% (n=86), from one to two, 17.7% (n=77), from three to five, 6.9% (n=30), from six to 10, 4, 2% (n=18), from 11 to 20 and 3.2% (n=14), 21 or more.

Table 2 presents the SAQ (by domains and overall) descriptive data. Only the total SAQ presented normal distribution and, therefore, it is described in mean and standard deviation. The domains presented abnormal distribution and are described according to median and breadth.

**Table 2:** Analysis of central tendency and variability measures and frequencies of positive safety culture or negative ratings of the Safety Attitudes Questionnaire (by domains and overall), Porto Alegre, Rio Grande do Sul, Brazil, 2018/2019 (n = 434)

SAQ	Mean	Standard deviation	Median	Minimum	Maximum	Negative safety culture n (%)	Positive safety culture n (%)
SAQ^*^ domains							
DOM1^†^ - Teamwork climate			70.83	0	100.00	243 (56)	191 (44)
DOM2 - Safety climate			64.28	0	100.00	294 (67.7)	140 (32.3)
DOM3 - Job satisfaction			80.00	0	100.00	188 (43.3)	246 (56.7)
DOM4 - Stress recognition			75.00	0	100.00	202 (46.5)	232 (53.5)
DOM5 - Perception of unit management			54.16	4.2	100.00	348 (80.2)	86 (19.8)
DOM6 - Perception of hospital management			50.00	0	100.00	357 (82.3)	77 (17.7)
DOM7 - Working conditions			58.33	0	100.00	270 (62.2)	164 (37.8)
Overall SAQ	63.14	13.92				353 (81.3)	81 (18.7)

* *SAQ - Safety Attitudes Questionnaire;*

† *DOM - Domain.*

Table 3 shows the statistical association between the safety culture classifications of the HSOPSC and SAQ instruments.

**Table 3:** Association between safety culture classifications by the Hospital Survey on Patient Safety Culture and the Safety Attitudes Questionnaire, Porto Alegre, Rio Grande do Sul, Brazil, 2018/2019 (n = 434)

		Overall SAQ ^†^		*p*
		Negative safety culture n (%)	Positive safety culture n (%)	
Overall HSOPSC^*^	Fragile areas of patient safety	247 (96.1) ^‡^	10 (3.9)	<0.001^§^
	Strong areas of patient safety	6 (17.6)	28 (82.4) ^‡^	

* *HSOPSC - Hospital Survey on Patient Safety Culture;*

† SAQ - Safety Attitudes Questionnaire;

‡ Associação estatisticamente significativa.

§ *Teste do Qui-Quadrado de Pearson.*

Regarding the magnitude of the correlation between the variables of the two instruments, the HSOPSC dimensions “DIM2 - supervisor/manager expectations & actions promoting safety” and “DIM5 - communication openness” had a high correlation with the SAQ domains “DOM1 - teamwork climate” (r=0.532 and r=0.563, respectively) and “DOM2 - safety climate” (r=0.527 and r=0.500, respectively), in addition to a moderate magnitude correlation with “DOM3 - job satisfaction” (r=0.378 and r=0.428, respectively), “DOM5 - perception of unit management” (r=0.442 and r=0.461, respectively), “DOM6 - perception of unit management” (r=0.442 and r=0.461, respectively), hospital management” (r=0.369 and r=0.371, respectively) and “DOM7 - “working conditions” (r=0.313 and r=0.306, respectively).

“DIM3 - organizational learning-continuous improvement”, “DIM4 - feedback and communication about error”, “DIM8 - hospital management support for patient safety”, “DIM9 - teamwork within Units” and “DIM10 - hospital handoffs & transitions” showed moderate correlation with “DOM1 - teamwork climate” (r=0.467; r=0.429; r=0.437; r=0.422; and r=0.398, respectively), “DOM2 - safety climate” (r=0.446; r=0.483; r=0.482; 0.403; and r=0.387, respectively), “DOM3 - job satisfaction” (r=0.432; r=0.334; r=0.486; r= 0.440; and r=0.340, respectively), “DOM5 - perception of unit management” (r=0.419; r=0.447; r=0.421; r=0.378; and r=0.326, respectively), “DOM6 - perception of hospital management “ (r=0.389; r=0.383; r=0.472; r=0.372; and r=0.338, respectively) and “DOM7 - working conditions” (r=0.328; r=0.337; r=0.416; r=0.309; and r=0.347, respectively).

“DIM1 - teamwork within units” and “DIM11 - perceptions of management” showed moderate correlation in relation to “DOM1 - teamwork climate” (r=0.448 and r=0.444, respectively), “DOM2 - safety climate” (r=0.472 and r= 0.426, respectively), “DOM3 - job satisfaction” (r=0.393 and r=0.368, respectively), “DOM5 - perception of unit management” (r=0.372 and r=0.357, respectively) and “DOM6 - perception of hospital management” ( r=0.330 and r=0.328, respectively). Finally, the other domains and dimensions did not present moderate to high correlations. When the general results of the two instruments were analyzed, a high correlation magnitude was observed, as shown in Table 4.

**Table 4:** Analysis of the overall perception of safety culture correlation between the Hospital Survey on Patient Safety Culture and the Safety Attitudes Questionnaire, Porto Alegre, Rio Grande do Sul, Brazil, 2019/2020 (n = 434)

	Overall HSOPSC^*^	Overall SAQ^**^
Overall HSOPSC^*^	1	0.564^‡^
Overall^†^ SAQ	0.564^‡^	1

* *HSOPSC - Hospital Survey on Patient Safety Culture.*

† SAQ - Safety Attitudes Questionnaire.

‡ *Pearson correlation test.*

## DISCUSSION

Among the participants in this study, there was a predominance of nursing technicians/assistants, corroborating several studies^(11,13,15-17,21,24-25)^ whose samples were composed mostly of nursing team professionals. Only in a multinational study with health professionals from 68 countries^(18)^, physicians prevailed in the sample.

According to the HSOPSC, less than 10% of participants had a strengthened perception of safety culture. “Teamwork within units” was the only dimension one that proved to be a strong area of patient safety, as in other studies^(15,26)^.

In the SAQ instrument, some studies show that “teamwork climate” reached the score for positive safety culture^(12,18,27)^, but in this study, the domain did not reach the median of 75 points, only approached. These results reflect a positive perception, since a good relationship between the team can have an influence on organizational climate, productivity, communication, care and health of workers^(12,24)^. Still, another Brazilian study pointed out that teamwork causes union and interaction between professionals when there is an overload of tasks to be performed quickly^(28)^.

“Nonpunitive response to error”, in the HSOPSC, was the one that obtained the lowest score, indicating that it is an extremely fragile area for patient safety in the institution, as well as in findings from a Brazilian study^(29)^, international studies^(11,13,15)^ and a study conducted in hospitals in Portugal and Brazil^(26)^. Another result, in relation to the question about the number of reporting, shows that almost half of participants in this study had not performed any in the last 12 months, since it was also present in several investigations^(13,15,20,26,28)^.

Analyzing the result that the dimension with the most fragile safety culture was “nonpunitive response to error” and that professionals had not been reporting safety incidents, it can be inferred that perhaps there was a fear that their errors would be used against them, instead of making an analysis to improve the processes and avoid their recurrence. This aspect portrays the existence of a punitive culture in the daily work process of nursing, which can be detrimental to the situational diagnosis and planning of patient safety actions, since it can lead professionals not to report on safety incidents and/or adverse events, or even to a false idea of safety^(28)^. Still on the HSOPSC, most workers assessed patient safety in their area/unit as “regular” as well as in another Brazilian study^(28)^.

In the safety culture assessment through the SAQ, the domains that reached the score of 75 points were “job satisfaction” and “stress recognition”, indicating a strong agreement of professionals regarding issues related to safety^(8)^. This result corroborates the findings of other Brazilian studies^(25,27,30)^, an Irish^(12)^ and a multinational^(18)^, in which “job satisfaction” also reached the score for positive safety culture.

On the other hand, there were studies in which no domain reached 75 points, such as one in Brazil^(24)^ and another in China^(14)^, but even so, “job satisfaction” obtained the highest score. However, although “stress recognition” is a domain of positive safety culture in this investigation, the result of no other study^(12,14,18,24-25,27,30)^ was similar. These data reflect that professionals were clear that factors such as tiredness, work overload and conflict situations are conditions that can impair the performance of their daily routines. Additionally, professionals’ satisfaction with their work is closely linked to the way they are heard by management and treated by other team members^(8)^.

“Perception of hospital management” and “perception of unit management” presented the lowest score, indicating a perception of negative safety culture, also found in other investigations^(18,24,30)^. The results are not satisfactory, reflecting the need to analyze possible improvements in relation to patient safety in Brazilian and international hospitals. A Brazilian study also pointed out the dissatisfaction of professionals with regard to hospital management activities, with regard to patient safety issues. This same study highlighted that management support is essential for the development of actions that allow for an improvement in the quality of care, such as the creation of a non-punitive safety culture, favoring the discussion of errors in order to improve processes^(24)^. The multinational study^(18)^, which had a predominance of medical professionals, also reported that closer collaboration between hospital management and health professionals may be one of the objectives for improving safety culture.

When analyzing the overall SAQ, professionals demonstrated a perception of negative safety culture. In other investigations^(14,24,30)^, the overall SAQ also did not reach the score of 75 points, considered satisfactory. There was a statistical association of fragile areas of patient safety (HSOPSC) with negative safety culture (SAQ) and, conversely, of positive safety culture (SAQ) with strong areas (HSOPSC). Furthermore, it is noteworthy that the “intermediate areas of patient safety” (HSOPSC) classification was closer to the positive safety culture (SAQ) classification. In this study, the SAQ overall presented a high magnitude correlation with the HSOPSC overall (r=0.564; p=<0.001), i.e., the two instruments converge for a similar assessment of patients’ perception of safety culture.

Although the findings are different from what was found in a Brazilian study^(20)^, both instruments presented high internal consistency according to Cronbach’s alpha. Investigation^(20)^ also revealed a weakened patient safety culture, presenting, as in this study, a statistical association (p <0.001) and high magnitude correlation (r=0.66); these values are close to those presented in the present investigation. It is noteworthy that the exclusion criterion “less than six months of admission” was present in both studies.

Unlike the results of this investigation, a Brazilian study^(20)^ found no correlation of high magnitude. However, in line with the results of this study, the authors also found a moderate association between “feedback and communication about error”, from the HSOPSC, and “safety climate” and “working conditions”, from the SAQ. Feedback to professionals about errors that occur in the unit, about the changes implemented from safety incident reports, as well as dialogues on how to prevent the recurrence of safety incidents, are fundamental and influence the safety climate, impacting communication on issues related to patient safety. Furthermore, an institution with this perspective tends to offer better working conditions to its employees.

In a study carried out in the United States of America^(17)^, which also assessed the correlation between the two instruments, “safety climate” presented a high magnitude of correlation with “perceptions of management” (r=0.72, p <0.001), unlike this investigation, in which “safety climate” correlated with high magnitude with “supervisor/manager expectations & actions promoting safety” (r=0.52) and “communication openness” (r=0.50). This same American study^(17)^ found a moderate magnitude correlation of the SAQ “teamwork climate” with the HSOPSC “teamwork within units” (r=0.67). In this investigation, a moderate correlation magnitude was also found between this SAQ domain and the HSOPSC dimension (r=0.42).

### Study limitations

Research in a single institution and only with nursing professionals is considered a limitation of this study.

### Contributions to nursing

This study contributes to the construction of evidence, which can be added to other existing ones with one or both instruments investigated, enabling comparative analyzes until we have more comprehensive studies in the country.

## CONCLUSIONS

It is concluded that the HSOPSC and SAQ instruments converge to a similar assessment of patient safety culture in the hospital environment, responding positively to the research question. This finding was also based on a high internal consistency in both instruments. “Nonpunitive response to error” assessment was highlighted as the most fragile area of patient safety, which corroborates low reporting in the last 12 months. Therefore, studies that analyze low compliance with safety incident reporting, whether due to fear of punishment, process failures or other factors, are necessary. In this context, nurses’ leadership role in managing processes and people is highlighted, with the aim of developing a non-punitive culture, encouraging reporting with an emphasis on learning and developing a patient safety culture.

The relevance of the results of this study lies in the identification that the two instruments used in Brazil, and in other countries, have a convergent assessment, which guarantees a more unified, rigorous and accurate analysis, regardless of which one is used, and it is even possible to compare the results with greater certainty between different hospitals.

Furthermore, it is suggested that health services invest in the development of a patient safety culture, with strategies for systemic knowledge of the gaps that lead to the occurrence of safety incidents, which can revert to safe environments, not only for patients, but also for professionals. It was identified, in this study and in others presented, that nursing has the “teamwork within units” as a strong area in the HSOPSC and that “job satisfaction” and “stress perception” in the SAQ reached the percentages required for a positive safety culture. Investigating the elements of these dimensions can be a way forward in the search for better performance to achieve appropriate health care, with permanent analysis of reality, mitigation of risks and incidents, to promote organizational development that prioritizes safe care.
